# Between Intent and Illness: A Look at Malingering vs. Factitious Behaviours

**DOI:** 10.1192/bjo.2025.10705

**Published:** 2025-06-20

**Authors:** Amy Anyi

**Affiliations:** Greater Manchester Mental Health Foundation Trust, Manchester, United Kingdom

## Abstract

**Aims:** Presented is a 33-year-old gentleman with a diagnosis of emotionally unstable personality disorder (EUPD) well-known to mental health services, including inpatient, community, liaison, and psychological care teams, with a long-standing history of self-harm and suicide attempts, which included deliberately placing himself in high-risk public areas which have at times resulted in detention under mental health legislation.

**Methods:** Over the past several years, this gentleman has fabricated claims of a cancer diagnosis, terminal prognosis, and multiple surgical procedures – assertions refuted by his medical records – while leveraging these falsehoods on social media and through a crowd sourcing campaign to raise funds by misrepresenting his physical health. Furthermore, he has strategically leveraged medical admissions to access medications, including strong analgesics and for a self-reported diagnosis that remains unverified.

During conducted assessments, he has expressed a desire for psychological therapy and enhanced crisis support yet consistently avoids engaging with the planned, regular support offered by teams who are familiar with his history, including appointments scheduled after episodes of self-harm.

While services have considered a factitious component in his presentation others contest it aligns more strongly with malingering. Consensus with professionals is that given his presentation there are difficulties in developing and maintaining a safe therapeutic relationship due to his disingenuity, threats of complaints, and his active avoidance of any meaningful, structured, recovery-focused work.

**Results:** Factitious disorder is driven by an internal need to assume the sick role and receive attention or care, with patients intentionally producing symptoms rooted in psychological need rather than for external rewards where the behaviour is characterized by a willingness to undergo invasive tests and treatments, reinforcing their patient identity. Factitious disorder is recognised as a psychiatric diagnosis warranting treatment, whereas malingering is motivated by external incentives and is not considered a mental illness but rather a behavioural strategy. Individuals who malinger tend to avoid procedures that might expose their deception and selectively engage in behaviours that yield tangible benefits.

**Conclusion:** This case underscores the importance of comprehensive, multidisciplinary assessments in achieving accurate diagnoses by clarifying key differences in motivation, behaviour, and clinical classification. Enhanced diagnostic clarity not only improves patient care, but also safeguards healthcare resources. Despite evident secondary gains in this case, the long-standing emotional instability and interpersonal dysfunction associated with EUPD still necessitate a balanced, empathetic therapeutic approach.

